# Sputum microbiota profiles of treatment-naïve TB patients in Uganda before and during first-line therapy

**DOI:** 10.1038/s41598-021-04271-y

**Published:** 2021-12-29

**Authors:** David Patrick Kateete, Monica M. Mbabazi, Faith Nakazzi, Fred A. Katabazi, Edgar Kigozi, Willy Ssengooba, Lydia Nakiyingi, Sharon Namiiro, Alphonse Okwera, Moses L. Joloba, Adrian Muwonge

**Affiliations:** 1 Department of Immunology and Molecular Biology, School of Biomedical Sciences, Makerere University College of Health Sciences, Kampala, Uganda; 2 BSL-3 Mycobacteriology Laboratory, Department of Medical Microbiology, School of Biomedical Sciences, Makerere University College of Health Sciences, Kampala, Uganda; 3 Department of Medicine, School of Medicine, Makerere University College of Health Sciences, Kampala, Uganda; 4 Infectious Diseases Institute, Makerere University College of Health Sciences, Mulago Hospital Complex, Kampala, Uganda; 5 Makerere University Lung Institute, Makerere University College of Health Sciences, Kampala, Uganda; 6 TB Clinics, Mulago National Referral Hospital, Kampala, Uganda; 7 Division of Genetics and Genomics, The Roslin Institute, University of Edinburgh, Edinburg, UK; 8 Division of Infection and Immunity, The Roslin Institute, University of Edinburgh, Edinburg, UK

**Keywords:** Microbiology, Molecular biology, Diseases, Medical research

## Abstract

Information on microbiota dynamics in pulmonary tuberculosis (TB) in Africa is scarce. Here, we sequenced sputa from 120 treatment-naïve TB patients in Uganda, and investigated changes in microbiota of 30 patients with treatment-response follow-up samples. Overall, HIV-status and anti-TB treatment were associated with microbial structural and abundance changes. The predominant phyla were *Bacteroidetes*, *Firmicutes*, *Proteobacteria, Fusobacteria* and *Actinobacteria,* accounting for nearly 95% of the sputum microbiota composition; the predominant genera across time were *Prevotella, Streptococcus, Veillonella, Haemophilus, Neisseria, Alloprevotella, Porphyromonas, Fusobacterium, Gemella*, and *Rothia*. Treatment-response follow-up at month 2 was characterized by a reduction in abundance of *Mycobacterium* and *Fretibacterium,* and an increase in *Ruminococcus* and *Peptococcus*; month 5 was characterized by a reduction in *Tannerella* and *Fusobacterium*, and an increase in members of the family *Neisseriaceae*. The microbiota core comprised of 44 genera that were stable during treatment. Hierarchical clustering of this core’s abundance distinctly separated baseline (month 0) samples from treatment follow-up samples (months 2/5). We also observed a reduction in microbial diversity with 9.1% (CI 6–14%) of the structural variation attributed to HIV-status and anti-TB treatment. Our findings show discernible microbiota signals associated with treatment with potential to inform anti-TB treatment response monitoring.

## Introduction

Tuberculosis (TB) is a persistent global public health problem and one of the top 10 causes of death worldwide^1,2^. Nearly half a million new TB cases have been reported in Uganda since 2010^1^, and the TB incidence in the country has surpassed that of HIV-infection^3^. While introduction of the Xpert MTB/RIF assay revolutionized the diagnosis of TB globally^4^, treatment still hinges on long treatment regimens i.e., 6–24 months depending on whether treating drug susceptible TB or drug resistant TB^5^. The standard first-line treatment regimen comprises of an intensive phase of 2 months treatment with isoniazid, rifampicin, pyrazinamide and ethambutol, followed by a continuation phase of 4 months treatment with isoniazid and rifampicin^6,7^. Following commencement of treatment with anti-TB drugs, sputum microscopy for identification of mycobacteria (in form of acid-fast bacilli, AFB) or where affordable, sputum culturing for *Mycobacterium tuberculosis* growth is done during the treatment period, usually at months 2 and 5 to monitor treatment response. Attaining sputum sterilisation, also known as sputum smear-conversion or sputum culture-conversion (i.e., from AFB smear/culture-positive to AFB smear/culture-negative) at months 2 or 5 after initiating treatment is a known cardinal index of treatment success. In this regard, the World Health Organization (WHO) guidelines state that “*a patient whose sputum was AFB smear-positive or culture-positive at the beginning of treatment but becomes smear-negative or culture-negative in the last month of treatment and on at least one previous occasion is declared cured*” of TB^6,8^. Despite the importance of sputum-smear/sputum-culture conversion in monitoring TB treatment response^9^, they have low sensitivity and in sub-Saharan Africa culturing is mainly done in reference/regional laboratories but not as routine. Therefore, identification of microbiological factors in a sputum sample, the cornerstone for diagnosing TB, and their association with anti-TB treatment response, could be useful in unravelling new approaches for improving treatment response monitoring^7^.

The microbiota are microorganisms—bacteria, fungi, protozoa and viruses that live on the skin and mucosa of humans and other mammals. Their role in induction, maintenance, disruption and modulation of the immune response has recently come into focus with the advent of the human microbiome project^10^. The microbiota also exist in the lung^11^, the predilection site for *M. tuberculosis* and perhaps influence its behaviour in a variety of ways—for example via signalling^11–13^, which could lead to positive interactions (synergism) and/or negative interactions (competition)^14^. Therefore, a sound understanding of the microbiota dynamics in TB is necessary given their emerging importance in human and animal health^11,15^. In this context, microbial profiling in TB could advance our knowledge of TB pathogenesis (infection vs. active disease) or unravel new ways in which TB diagnostics and management can be improved^7,11–13^. Although microbiota shifts have been associated with several conditions and infectious diseases including pulmonary TB^11,12,16,17^, there is a general lack of knowledge on microbiota and disease in sub-Saharan Africa^11^ where there is high burden of both TB and HIV.^18^ For the first time, we report the sputum microbial composition of treatment-naïve TB patients in Uganda, and the impact of first-line anti-TB drugs on the microbiota using sputum as proxy for the lung environment^19^. As well, we describe the microbial changes associated with critical transitions of anti-TB treatment: pre-treatment (baseline) and treatment response follow-up at months 2 and 5.

## Results and discussion

### Patients and samples

We enrolled 120 treatment-naïve TB patients at Mulago National Referral Hospital (Mulago hospital) in Kampala, Uganda, in the period between 2016 and 2018. Table 1 summarises the clinical and demographic characteristics of the patients; the mean age was 33 years—majority were male and residents of greater Kampala metropolitan area (i.e., Kampala city proper and the neighbouring districts of Wakiso, Mukono, Mpigi, Buikwe and Luweero), Supplementary Fig. S1 online.

**Table 1:** Clinical and demographic characteristics of pulmonary TB patients enrolled.

Variable	Patients, n = 106^a^ (%)	Proportion by gender (%)	
		Female, n = 38 (36)	Male, n = 68 (64)
**HIV status**			
Positive	23 (22)	10 (44)	13 (57)
Negative	83 (78)	28 (34)	55 (66)
**Chest X-ray**			
Cavities	69 (65)	25 (36)	44 (64)
No cavities	37 (35)	13	24
**Medication**			
HIV-positive on ART	16 (60)	8 (50)	8 (50)
History of antibiotics use	22 (18.3)	8 (36)	14 (64)
Others^b^	23 (19.2)	11 (48)	12 (52)
None reported	45 (42)	11	34
**Comorbidity**			
Hypertension	10 (9)	2 (20)	8 (80)
Smoking	26 (25)	1 (4)	25 (96)
Consume alcohol	40 (38)	11 (28)	29 (73)
None reported	30 (28)	24	6
**BMI**			
Low (≤ 18)	59 (56)	19 (32)	40 (68)
Medium (≥ 19–25)	43 (41)	16 (37)	27 (63)
High (≥ 26–29)	4 (4)	3 (8)	1 (2)
**Highest level of education**			
None	6 (6)	5 (83)	1 (17)
Primary	21 (20)	9 (43)	12 (57)
Secondary (O’ / A’ levels)	79 (75)	24 (30)	55 (70)
**District of residence**			
Kampala	61 (58)	24 (39)	37 (61)
Wakiso	35 (33)	12 (34)	23 (66)
Others^c^	10 (9)	2 (20)	8 (80)
**Ancestry**			
Bantu	92 (87)	30 (33)	62 (67)
Non-Bantu	14 (13)	8 (57)	6 (43)
**Employment status**			
Employed	94 (89)	31 (33)	63 (67)
Not employed	12 (11)	9 (75)	3 (25)
**Conventional TB diagnostics**			
*AFB*			
No AFB	16 (15)	7 (44)	9 (56)
Scanty	17 (16)	6 (35)	11 (65)
1 + AFB	10 (9)	2 (20)	8 (80)
2 + AFB	29 (27)	12 (41)	17 (59)
3 + AFB	34 (32)	11 (32)	23 (68)
*Xpert MTB/RIF*			
Positive	106	35	71
Negative	NA	NA	NA
*LJ culture*			
Positive (%)	83 (78.3)	23 (28)	60 (72)
Negative (%)	23 (22)	15 (65)	8 (35)
**Sampling point (month)**			
0 (baseline before treatment)	106 (100)	46 (43.4)	60 (56.6)
2 (1st treatment response follow-up)^d^	44 (41.5)	15 (34.1)	29 (65.9)
5 (2nd treatment response follow-up)^c^	41 (38.7)	16 (39)	25 (61)

*ART* antiretroviral treatment, *AFB* acid fast bacilli, *BMI* body mass index, *LJ* Lowenstein-Jensen medium, *NA* not applicable.

^a^Fourteen baseline samples were removed due to poor sequence quality leaving 106 patients (one sample per patient) with sequences that were analysed (120 − 14 = 106).

^b^Refers to medicines other than antibiotics and antiretrovirals (ARVs).

^c^Refers to participants staying in districts other than Kampala and Wakiso.

^d^Of the 106 patients enrolled and sampled at baseline, the available samples from treatment response monitoring at months 2 and 5 represented 30 patients i.e., 44 and 41, respectively.

### Taxonomic composition of the sputum microbiota

High-throughput sequencing of the variable region of the *16S rRNA* gene generated a total of 9,316,821 sequence reads from the 205 sputum samples i.e., 120 (baseline), 44 (month 2), and 41 (month 5). After filtering and quality control, we retained 8,638,640 sequences representing 191 samples—106 (baseline), 44 (month 2), and 41 (month 5) that were analysed; 14 baseline samples were removed due to poor sequence quality, Table 1. The retained high-quality sequences yielded 8,180 operational taxonomic units (OTUs), 18 phyla and 333 genera.

**Figure 1:**
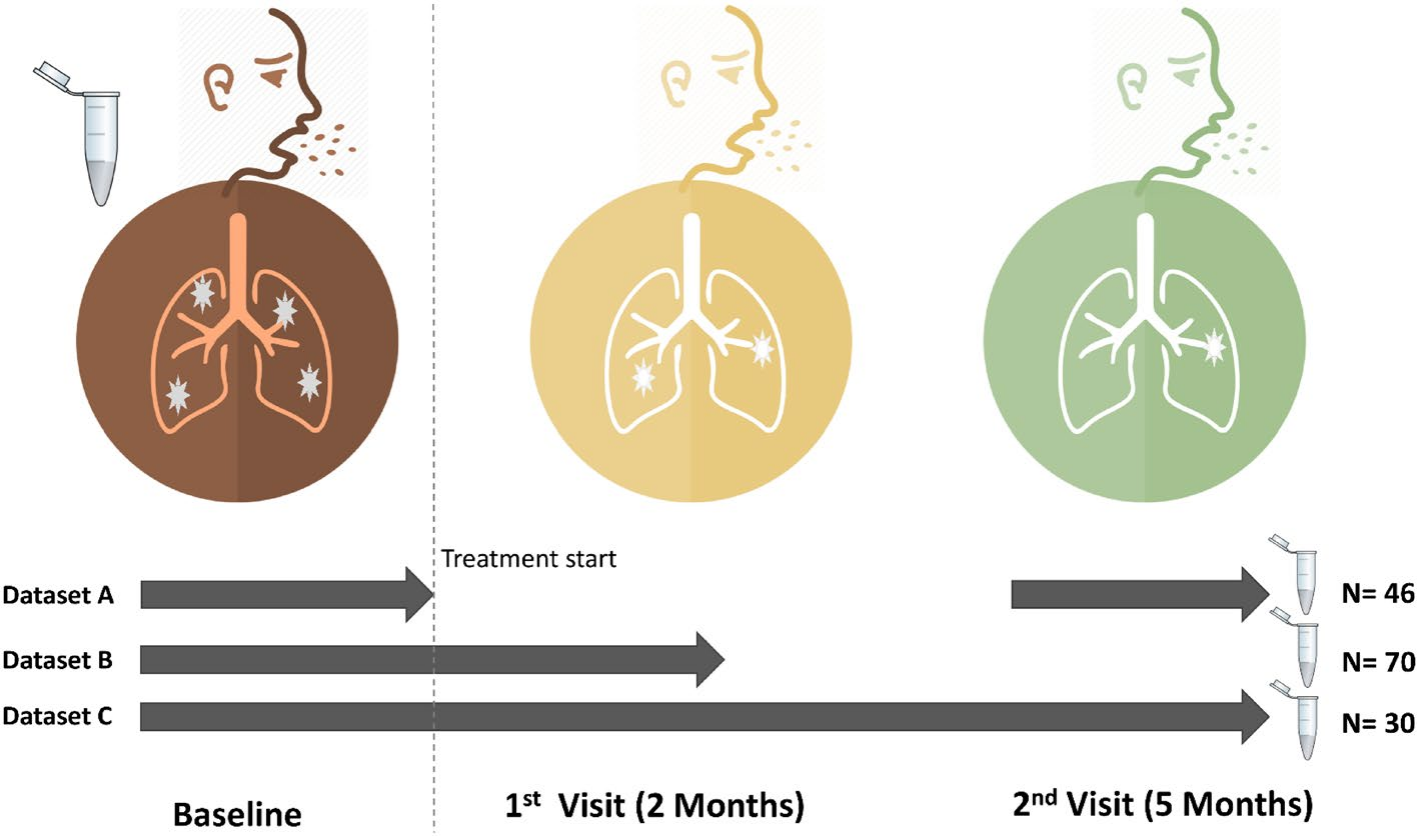
Participants’ enrolment, sampling and treatment response follow-up at months 2 and 5. A total of 120 pulmonary TB patients were enrolled at month 0 (baseline) prior to treatment commencement with first-line anti-TB drugs; of these, 70 had sputum samples at months 0 and 2 (dataset B), 46 had samples at months 0 and 5 (dataset A), while 30 had samples at months 0, 2 and month 5 (dataset C). The microbiome analyses reported in this study focus on dataset C. Dark red, orange and green icons depict months 0, 2 and 5, respectively.

Figure 1 shows the sputum samples collected from patients at baseline (i.e., month 0 prior to anti-TB treatment commencement) and treatment response follow-up visits (months 2 and 5). Of the 106 patients enrolled, 30 had samples across the three-sampling points – Fig. 1, dataset C; 70 had samples at baseline and month 2 (dataset B), 46 had samples at baseline and month 5 (dataset A)—the microbial analyses reported in this study focus on dataset C to allow for within and between patient comparison of microbiota changes. However, to fully explore the breadth and depth of microbial characteristics for TB patients, we explored the entire dataset (Fig. 1) and show that a sputum sample on average generated 44,992 sequences, 6,580 OTUs, 4 phyla and 36 genera (also see Supplementary Figs. S2, S3 and S4). The predominant phyla detected were *Bacteroidetes, Firmicutes, Proteobacteria, Fusobacteria* and *Actinobacteria*, accounting for nearly 95% of the sputum microbiota composition.

**Figure 2:**
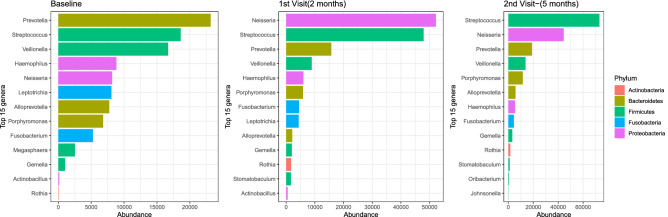
The 15 major bacterial genera detected across the three-sampling points.

While we observed similar microbial composition at phylum level in the sputa of 30 patients with samples across the three-sampling points, the composition of the bacterial genera in treatment follow-up samples varied compared to baseline samples, Fig. 2. We observed a dramatic reduction in number of sequences mapping to the genus *Mycobacterium* at month 2, probably due to the effect of anti-TB drugs. Overall, 10 major genera—*Prevotella, Streptococcus, Veillonella, Haemophilus, Neisseria, Alloprevotella, Porphyromonas, Fusobacterium, Gemella* and *Rothia*, were detected across the sampling period. *Megasphaera* was detected only at baseline (month 0); *Stomatobaculum* was absent at baseline but occurred at months 2 and 5. Furthermore, *Leptotrichia* and *Actinobacillus* were present at baseline and month 2 but undetected at month 5, while *Oribacterium* and *Johnsonnella* exclusively occurred at month 5, Fig. 2. Overall, 617 OTUs and 91 genera were shared between patients across the three-sampling points. The changes in abundance of some of the dominant genera i.e., *Megasphaera*, *Stomatobaculum, Leptotrichia* and *Johnsonnella* could represent microbial shifts worth exploiting to improve monitoring of TB treatment in the future.

Although sputum microbial characteristics of pulmonary TB patients have been explored before^11,17,20,21^, for the first time we report on this from a country where HIV-associated TB is prevalent^1,22^ and ranked by the WHO alongside 29 other countries as having the highest burden of TB globally^1^. Overall, our study generated nearly 16 times the number of OTUs reported in Asia^16,17^, probably due to differences in amplicon sequencing technology used i.e., MiSeq/illumina sequencing platform vs. 456/pyrosequencing in previous studies. Equally, these differences may signify a higher sputum microbiota diversity for Ugandan TB patients: indeed, the microbiota diversity observed in this study accounts for nearly 48% of the diversity in the human oral microbiome database http://www.homd.org. Generally, the sputum microbiota composition of pulmonary TB patients has been previously described as complex and more diverse than that of healthy control participants^23^, and our report seems to conform to this trend i.e., the sputum microbiota of TB patients in Uganda appears as complex and diverse as reported for TB patients in Asia^16,17,23,24^. For example, there was an average of 6580 OTUs and 36 genera in a sputum sample in this study vs. 602 OTUs and 12 genera in a sputum sample in India^17^. Although at high taxonomic level (i.e., phylum) the microbiota profile of TB patients in Uganda is comparable to the one reported in previous studies (e.g., the five major taxa described in this study—*Actinobacteria*, *Bacteroidetes*, *Firmicutes*, *Fusobacteria* and *Proteobacteria,* were previously reported as major phyla in Asia comprising of ≥ 98% of the sputum microbiota composition of pulmonary TB patients^16,17,23^), genus level analysis reveals considerable differences in diversity and distribution of the major genera detected in Ugandan vs. Asian TB patients^16,17,23,24^ i.e., *Prevotella, Streptococcus, Veillonella, Haemophilus, Neisseria, Alloprevotella, Porphyromonas, Fusobacterium, Gemella*, *Rothia, Leptotrichia*, *Megasphaera*, *Stomatobaculum*, *Oribacterium*, and *Johnsonella*. *Prevotella*, *Streptococcus* and *Veillonella* are the three most prevalent genera in this study, which is in agreement with the findings of a previous study in China^16^; however in India, one study reported *Streptococcus*, *Neisseria* and *Veillonella* as the three most prevalent genera^17^. Of note, *Prevotella* is consistently reported as a major component of sputum microbiota in chronic obstructive pulmonary disease (COPD), cystic fibrosis, pulmonary TB and lower respiratory tract infections^16,23^. Furthermore, while genera like *Leuconostoc*, *Lactobacillus, Corynebacterium*, *Bacillus*, *Acinetobacter, Granulicatella*, *Actinomyces*, *Anoxybacillus*, *Klebsiella*, *Pilibacter*, *Abiotrophia*, *Paucisalibacillus, Stenotrophomonas*, *Cupriavidus*, *Pseudomonas*, *Thermus*, *Sphingomonas*, *Brevundimonas*, *Brevibacillus*, *Methylobacterium*, *Diaphorobacter*, *Comamonas*, *Mobilicoccus*, *Gramulicatella* and *Fervidicoccus* were described as prevalent and abundant in pulmonary TB patients in India^17^ and/or China^23,24^, they are not among the 15 major genera reported in our study. These differences likely represent inherent environmental characteristics between the two geographic locations; the granular role on TB treatment outcomes is beyond the scope of this study but merits further study.

### A comparison of amplicon sequencing and conventional TB diagnostics

In this study, there was a 0.61 (CI 0.50–0.71) and 0.63 (CI 0.51–0.73) agreement between the detection of *Mycobacterium* sequences and positive outcome on microscopy and culture (Löwenstein-Jensen—LJ), respectively. In other words, detection of *Mycobacterium* sequences in a microbiome dataset correlated with sputum-smear positivity and/or sputum-culture positivity; a Kappa test for agreement showed a 61% agreement between amplicon sequencing and microscopy, and a 63% agreement between amplicon sequencing and culture. However, this potential diagnostic utility i.e., high positive predictive value of 0.71 (CI 0.59–0.81) and 0.75 (CI 0.63–0.91) respectively, is eroded by poor specificity (see Supplementary Table S1 and Supplementary Fig. S2 online). Therefore, while amplicon sequencing ably identified smear-positive and/or culture-positive TB patients, its performance with smear-negative and/or culture-negative TB samples was poor. Furthermore, while amplicon sequencing is 10 times more expensive than microscopy (e.g., the cost for amplicon sequencing is $25 [US dollars] per sample vs. ≤ $2.5 per sample for microscopy), it is considerably less expensive than culture (e.g., the cost for TB culture in resource-limited settings is on average $62.1 per sample^25^). Therefore, amplicon sequencing could be a good diagnostic alternative in resource-poor settings where culture is very expensive.

We also note that smear-negative and culture-negative samples produced comparatively more sequence reads than the smear-positive and culture-positive samples (see Supplementary Table S1 and Supplementary Fig. S2 online). This was a consistent observation for which the implication is currently not clear but could be attributed to low yield of the extracted total DNA from the smear-positive and culture-positive samples relative to the smear-negative and culture-negative samples. On the other hand, as respiratory infections are caused by bacteria and viruses that frequently interact with each other (reviewed by Bosch et al.^14^), perhaps growth/presence of *M. tuberculosis* is antagonistic to growth/presence of certain bacteria or vice-versa. However, this notwithstanding, there was an expected concordance between the *Mycobacterium* sequence count and colony forming units (CFU) on LJ cultures i.e., the sputum culture negative samples with higher total bacterial sequence reads also yielded culture-negative results.

### Sputum microbial diversity

**Figure 3:**
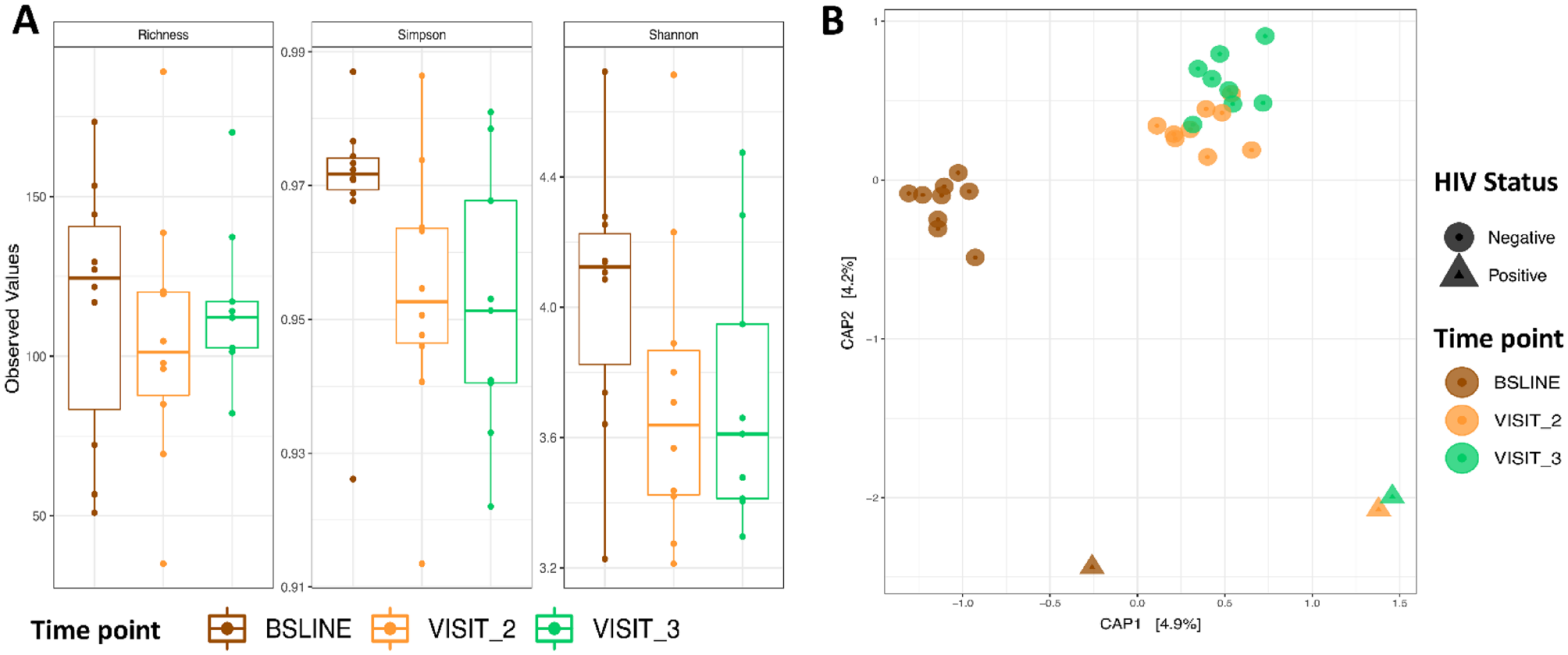
Microbial diversity analysis during first-line anti-TB treatment. (**A**) Depicts Alpha diversity for indices like Richness, Simpson and Shannon. (**B**) Depicts Beta diversity based on constrained ordination of the Bray–Curtis distances. Brown, orange and green represent months 0 (baseline), 2 and 5, respectively. The circular and triangular shapes in (**B**) denote HIV-negative and HIV-positive, respectively. Note this figure is based dataset C of Fig. 1 (n = 90 samples, 30 from each sampling point).

To understand the impact of anti-TB treatment on microbial structure, we analysed dataset C (Fig. 1) that depicts the 30 patients with samples across the three sampling points – baseline, months 2 and 5. The impact on microbial structure was determined by assessing Alpha and Beta diversities, all shown in Fig. 3: In brief, we observed a significant difference in microbiota diversity in treatment follow-up samples (collected at months 2 and 5) compared to baseline samples (collected before initiating anti-TB treatment) at alpha diversity level on indices like Richness, Simpson and Shannon, Fig. 3A. The largest change in diversity was observed between baseline and month 2 samples, which highlights the impact of TB treatment on the microbial community and specifically the genus *Mycobacterium* as noted above. The constrained ordination in Fig. 3B (and Fig. 7B) shows the microbial structural changes during treatment, here patients clustered by HIV status (i.e., HIV-positive vs. HIV-negative) and sampling point (baseline vs. months 2 and 5), explaining 9.1% of the variance. Indeed, the PERNANOVA multivariable analysis indicates that TB treatment accounts for 4–6% of the total effect-size on the microbial structure shown in Supplementary Table S2 (online) and Supplementary Fig. S3 (online) i.e., body mass index (BMI) (4%, 3%,11%), nutritional status (3.5%, 0.5%, 0.7%), and HIV status (4%, 3.4%, 1.5%) changes in Bray–Curtis, Weighted and unWeighted unifrac distances, respectively. These variables cumulatively explain 9.4%-13.1% of the total variance i.e., microbial structural shift across the sampling period. Supplementary Fig. S5 (online) depicts two bacterial families with the highest variability relative to HIV status while Supplementary Fig. S6 (online) depicts the microbial diversity of all patients regardless of visits.

Since a large proportion of the explained variance was attributed to sampling point, probably a significant decrease in the *Mycobacterium* reads at months 2 and 5, as expected from anti-TB treatment, could be producing this signal. Indeed by month 2 there was a general reduction in total microbiota abundance, which could be attributed to the administering of rifampicin, an antibiotic with a broad-spectrum of activity^11^. Even after controlling for factors like sex, HIV status and BMI, this marked change in abundance remained. However, from the clustering depicted in Fig. 3B (and Fig. 7B), the microbiota grouped patients not only according to their clinic visits but also according to their HIV- and nutritional status hence, other factors^26^ also contribute to the observed signal and these can be investigated in future for an insight into anti-TB treatment response monitoring for example, by examining the characteristics of patients with group-specific and/or stage-specific bacterial families/genera e.g., *Stomatobaculum*, *Oribacterium* and *Johnsonella* that increased during treatment and were missing at baseline (see Supplementary Fig. S5 online). Overall, the differential clustering of patients in this study contrasts with reports from Asia, in which no obvious clustering of TB patients was observed^16^ but it is important to note that unlike this study, all the TB patients in Asian studies were HIV-negative^16,17,24^.

### Core and accessory microbiota

**Figure 4:**
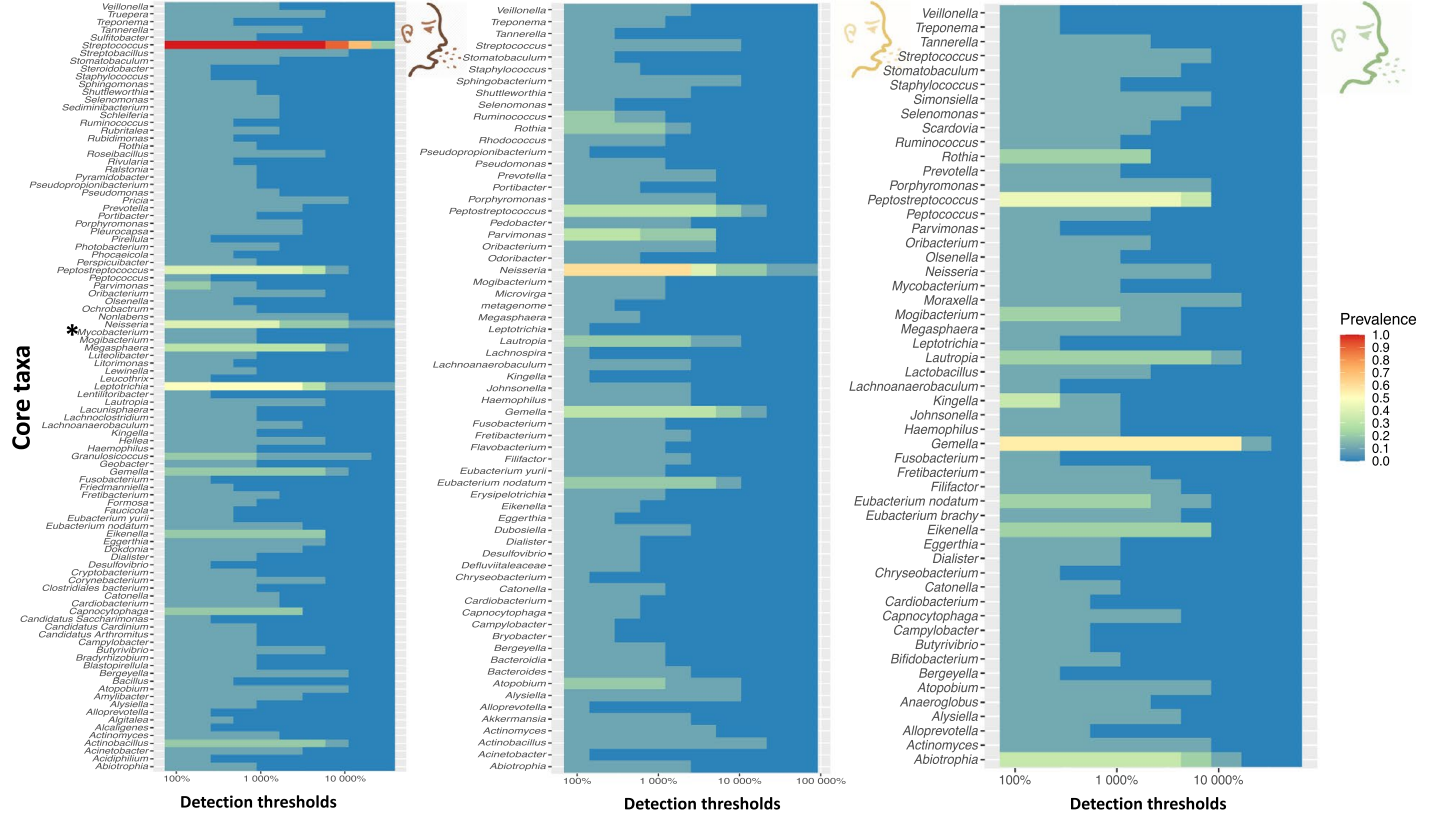
Characterizing the core microbiota during anti-TB treatment. The three panels depict composition of the core genera during treatment as defined by QIIME-2 microbiome package. Note that the genus *Mycobacterium* is a member of the core before initiating treatment but not during/after treatment. This analysis is based dataset C of Fig. 1.

**Figure 5:**
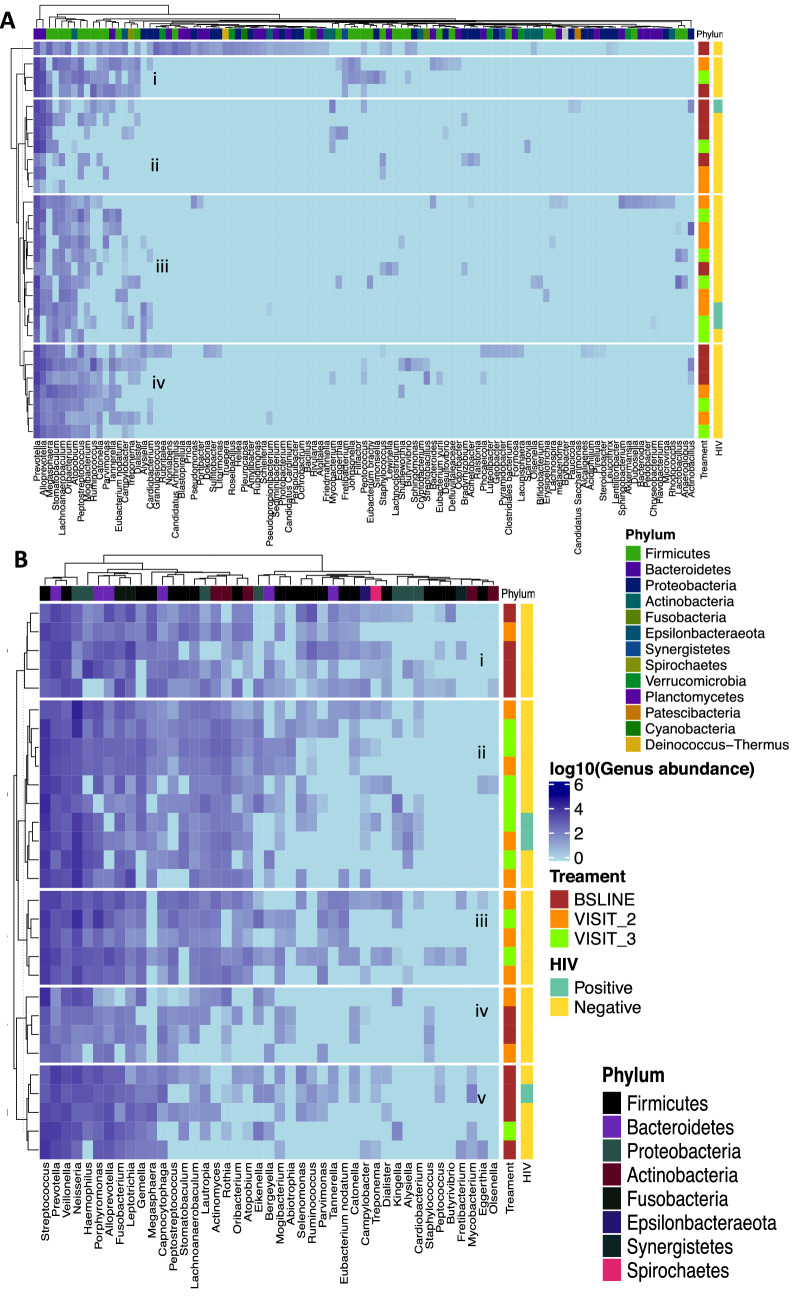
Hierarchical clustering of the normal flora (**A**) and core (**B**) at genera level. This clustering allows us to identify groups (i)–(v) that correspond to clinical covariates i.e., treatment response follow-up time and HIV status. This figure is based on dataset C of Fig. 1.

To examine how the microbial components are affected by anti-TB treatment, we analysed dataset C in Fig. 1 and tracked components defined as ‘core’ (i.e., the genera present in eighty percent of the samples at a given sampling point^27^—in this case baseline, months 2 and 5) and ‘accessory’ (the difference between the core and richness, which provides insight into the characteristics of the transient microbiota). We characterised and tracked the size and composition of core and accessory microbiota in sputum samples (n = 90) of 30 patients collected at baseline (month 0) and treatment follow-up visits (months 2 and 5), Figs. 4 and 5, respectively. The change in abundance of core members is shown in Fig. 5B; interestingly, the core size was remarkably stable with an average of 44 genera over the sampling period. It is noteworthy that *Mycobacterium*’s membership of the core was restricted to baseline samples before commencement of anti-TB treatment (Fig. 4). Indeed, at baseline before initiating treatment, *Mycobacterium* was a member of the core accounting for 1 in 500 bacteria in sputum. By months 2 and 5, the proportion of *Mycobacterium* fell to 1 in 5000 and 1 in 10,000, respectively, and its membership shifted to the accessory microbiota. This observation is in line with the clustering of patients by Ziehl–Neelsen (ZN) sputum smear status therefore, it supports sputum as a sample of choice for anti-TB treatment response monitoring.

**Figure 6:**
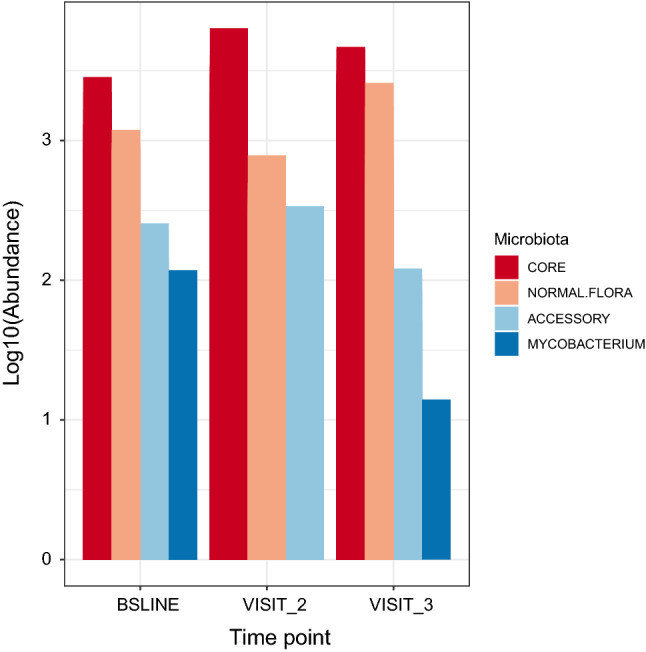
Changes in microbial components i.e., core and accessory microbiota; oral disease associated microbiota and *Mycobacterium*. This figure is based database C of Fig. 1.

The ‘core’ comprised of genera like *Streptococcus*, *Veillonella*, *Neisseria*, *Fusobacterium*, *Lachnoanaerobaculum*, *Atopobium*, *Peptostreptococcus* and *Leptotrichia*. Of these, *Streptococcus* was the most abundant but unlike *Veillonella*, its abundance was consistent before and after initiating treatment. Conversely, genera like *Neisseria* showed a steady increase over time. Figure 6 shows that most of the core genera are normal flora hence the similar patterns across sampling points. Further investigation of the abundance of each of the microbiota components showed subtle differences; clusters like (i) and (v) in Fig. 5 mapped to treatment groups while (iv) and (v) had a comparatively depleted core. Although not exclusive, clusters (i) and (iv) were predominated by patients who had started anti-TB treatment. Figure 5A examines the signatures of change in normal flora that appear to be less discriminatory as no cluster exclusively maps to a time point.

Core microbiota as characterised in this study is a close approximation of the normal flora, a significant contributor to maintenance of the integrity of several anatomical sites including the oral cavity and lung^11^. Dysbiosis (a deviation from normal flora) has been associated with dysregulation of the immune response, which alters the environment in favour of invading/foreign bacteria^10^; we noted a high biomass of the accessory microbiota, which supports the notion that dysbiosis promotes proliferation of bad/foreign bacteria^10^ e.g., *Actinobacillus, Bergeyella* and *Fretibacterium* that were components of the accessory microbiota. Overall, these findings suggest that the accessory microbiota could also be playing a critical role in augmenting sputum microbiota dynamics but the pathobiological implication of this remains unclear.

### Sputum microbial abundance and clinical covariates

**Figure 7:**
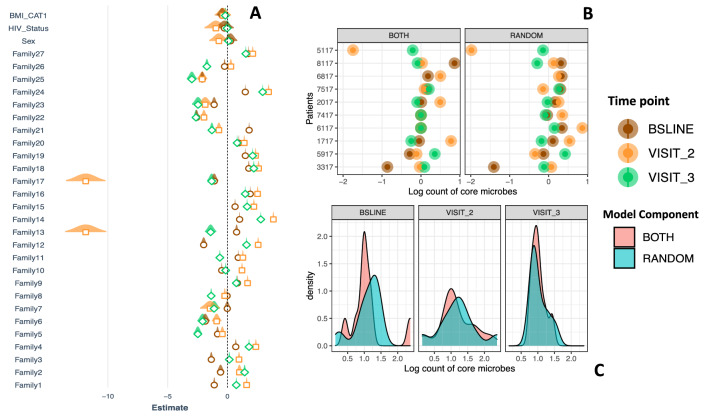
Analysis of microbial-abundance. (**A**) Depicts change in abundance across the treatment period accounting for BMI, HIV status and gender. Brown, orange and green depict months 0 (baseline), 2 and 3, respectively. (**B**) depict the variance attributed to an individual sample (random intercept of our mixed model) and that due to clinical variables (fixed component of the model), the latter was larger than the former, especially between months 0 (baseline) and 2 (**C**). This figure is based dataset C of Fig. 1.

To quantitatively investigate the impact of treatment, we used a Poison mixed effects regression model, with the abundance of bacterial families as the outcome variable. Figure 7 shows the fixed and random components in panels A, B and C, respectively. We observed a dramatic change (~ 9 log reduction) in the abundance of families such as *Peptostreptococcaceae* (#13) and *Ruminococcaceae* (#17) at month 2, panel A. Other families like *Streptococcaceae* (#1), *Leptotrichiaceae* (#12), *Lachnospiraceae* (#14), *Erysipelotrichaceae* (#24) and *Campylobacteraceae* (#21) significantly changed between the three sampling points. Interestingly, when we account for the variation due to our explanatory variables, the change in abundance of *Mycobacterium* observed before becomes less dramatic. Generally, the model based on dataset A shows inherent variations in abundance of core microbes but within this, anti-TB treatment associated changes are discernible, panel A. The random component of this model (Fig. 7B,C) suggests that an individual sample from a TB patient accounts for, on average, 0.27 (se = 0.52), 0.53 (se = 0.73) and 0.04 (se = 0.21)) of the variation but this changes to 0.18 (se = 0.42), 0.41 (se = 0.64), 0.03 (se = 0.18) respectively, when we account for the fixed effects component at the three sampling points. Therefore, clinical covariates explain most of the variance at baseline and month 2 whilst most of the variation is explained by the sample at month 5, Fig. 7B,C.

**Figure 8:**
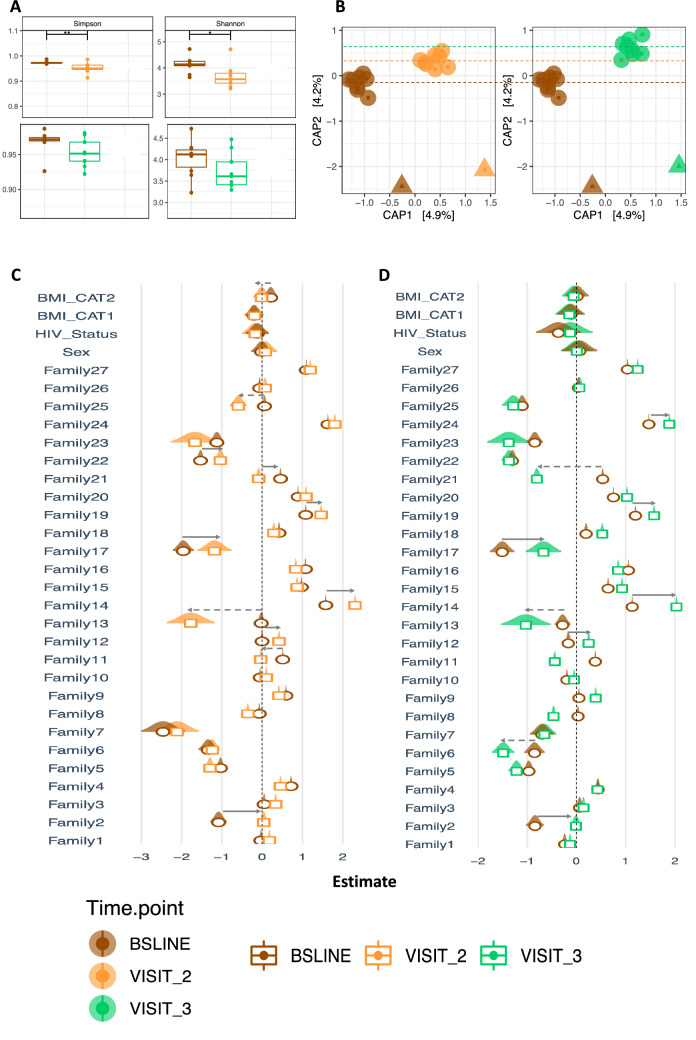
Analysis of the magnitude and direction of change relative to baseline. This figure is based database C of Fig. 1.

Figure 8 shows the changes in microbiota composition relative to baseline samples. Here, we observe a significant difference in the Shannon and Simpson diversity indices between the baseline and first treatment follow-up visit (month 2) but not the second treatment follow-up visit (month 5), panel A. We also observe distinct clustering by treatment follow-up period on the constrained ordination analysis, panel B. We note that BMI is associated with microbial abundance shifts between baseline and month 2, characterised by a reduction in abundance of genera like *Mycobacterium* (#25), *Olsenella* (#13) and *Haemophilus* (#11), and an increase in genera/families like *Ruminococcus* (#22) and *Lachnospiraceae* (#12) at month 2 relative to baseline, panel C. At Month 5, there was a reduction in genera like *Tannerella* (#21), *Atopobium* and *Olsenella* (#13), and an increase in *Haemophilus* (#14), *Peptostreptococcus* (#17), *Ruminococcus* (#19), *Mycobacterium* (#24) and core members of the families *Neisseriaceae* (#2) and *Lachnospiraceae* (12), panels C and D.

Finally, we acknowledge certain limitations in this study: first, it is difficult to normalise for sputum collection and processing, which has the potential to introduce variations. Using sputum to represent the lung environment only provides an approximation—we therefore recommend a comparison with bronchoalveolar lavage (BAL) samples, as well as stool to investigate the emerging pathogenic associations between lung function and gut microbiota (the ‘gut-lung axis’)^28^. Second, we did not include samples from healthy individuals as controls to compare with the microbiota from TB patients. It is noteworthy including such controls comes with complicated ethical issues; nonetheless, a longitudinal study allowed us to account for within and between patient dynamics across time. In reality, studies that have used sputum microbiota of TB patients and age-matched healthy controls^15,16,20,21^ suggest limited utility given the similarities observed.

## Conclusions

In a TB endemic setting with high prevalence of HIV/AIDS, sputum microbiota parameters of treatment-naïve TB patients map to patient characteristics regarding clinic visits, HIV and nutritional status. Moreover, the sputum microbiota core size does not change during treatment; in contrast, the accessory microbiota to which *Mycobacterium* belongs, changes significantly. Taken together, the changes in microbial structure, microbial components (core and accessory), attributable variance and magnitude and direction of change suggest that treatment with first-line anti-TB drugs has a significant impact on sputum microbiota, which is largest at month 2 of treatment response follow-up. This study demonstrates the potential utility of clinical metagenomics as diagnostic approach to inform anti-TB treatment response monitoring in resource-limited settings where culture remains expensive.

## Methods

### Study setting

This longitudinal study was conducted at Mulago hospital in Kampala, Uganda, between 2016 and 2018. Mulago is the largest public hospital in Uganda with 1500 beds, a TB clinic and a multidrug resistant (MDR)-TB treatment centre for the country. Around 5000 TB patients are treated at the clinic every year, of whom one third are retreated patients^29^. We randomly enrolled 120 treatment-naïve TB patients 18 years and older who were confirmed to have pulmonary TB, collected sputum and profiled them (i.e., collecting and organizing clinical and demographic data in a way that enables its mapping to the microbiota) before initiating anti-TB treatment which formed the baseline for the study. Pulmonary TB disease was clinically diagnosed by Pulmonologists at the TB clinic and confirmed with the Xpert MTB/RIF assay on sputum; however, ZN staining for AFB detection and LJ culturing was performed for all samples at the BSL-3 Mycobacteriology Laboratory, Makerere University College of Health Sciences; the Mulago Hospital TB clinics and Makerere University laboratories are located on the same campus (for sample work-flow see Supplementary Fig. S7 online). Additional samples from treatment-response follow-up visits (at months 2 and 5) were available for 30 patients; these were compared to baseline samples to unravel the microbiota changes and dynamics after initiating treatment. To account for potential cofounders, we included a wide range of clinical and lifestyle variables.

Sputum samples were collected with consent as part of routine clinical care however, to ensure consistency in quality and quantity of the sputum collected, sputum induction was performed by an expert Pulmonologist at the TB clinic as described previously^30^. Specimen containers with sputum were tightly closed and placed in plastic biohazard bags and immediately transported to the BSL-3 Mycobacteriology laboratory in ice-cool boxes, where the samples were processed as previously described^31^ for AFB detection and culturing on LJ medium. Briefly, to 5–10 ml of specimen, an equal volume of a mixture of NALC-NaOH at a concentration of 3% NALC and 6% NaOH was added, vortexed for 5 min and incubated at room temperature for 15 min. The digested sample was diluted to the 50 ml mark with phosphate buffer (pH 6.8), mixed thoroughly and centrifuged at 4000*g* for 15 min. The sediment was adjusted to 2.5 ml with sterile phosphate buffer saline (PBS); 0.5 ml of the suspension was used to inoculate LJ slants. A portion of the sputum sediment (in ~ 0.5 ml) was sent to the Molecular Diagnostics laboratory, adjacent to the BSL-3 Mycobacteriology laboratory for molecular analysis. Chromosomal DNA was extracted from the pellets by using the AllPrep DNA/RNA Mini Kit (Qiagen) according to the manufacturer’s instructions.

### DNA sequencing and sequence analysis

High throughput sequencing of sputum DNA targeting the V3-V4 region of the *16S rRNA* gene was performed at the Makerere University Genomics/Molecular Diagnostics Laboratory and the Integrated Microbiome Resource (IMR), Dalhousie University Canada (https://imr.bio) ^32,33^ on a MiSeq platform (illumina Inc.). Sequence reads were processed by using the Quantitative Insights into Microbial Ecology version 2 software (QIIME-2)^34^. Briefly, raw paired-end sequence reads and metadata were combined to generate a QIIME-2 object (artefact) for subsequent analysis^34^. Paired sequence reads were filtered for quality, dereplicated, chimeras removed and denoised by using DADA2^34^. This generated amplicon sequence variant (ASV) tables (previously known as operational taxonomic unit [OUT] tables) and representative sequences (in this study, the terms “ASVs” and “OTUs” are used interchangeably). The OTU tables were used to estimate Alpha and Beta diversity indices at an OTU minimum depth threshold of 2000. This was followed by a step to identify potential contamination. Alpha diversity indices included observed OTUs and Shannon^35^. Beta diversity was estimated by using Bray–Curtis, weighted and unweighted uniFrac distances^34^. Phylogenetic analysis was inferred with phyloseq package v1.26.0^36^ and Metacoder v0.3.2-based microbiome analysis in R statistical package (v3.5.1)^15^ during beta diversity estimation. This was done by aligning representative sequences, filtering out non-informative sites and generating a rooted tree using MAFFT and FASTTREE^34^ software. The taxonomic classification of OTUs was achieved by using a naïve Bayes classifier trained on the most recent SILVA database at 97% similarity^37^. First, the training dataset was extracted by using primers used for sequencing the samples^32^, and the resultant dataset used to train the classifier for taxonomically assigning the OTUs. Visualisation of taxonomic abundance was done by using ggplot2 in R.

### Microbiota community structure and clinical covariates

To assess the explanatory power/effect size of variables for changes in the microbiota community structure, we used sampling point at months 2 and 5 as proxy for monitoring the effect of anti-TB treatment on the microbiota. We performed a constrained analysis of distance-based redundancy (CAP); in this case we used Bray–Curtis as the outcome variable, HIV status and sampling point as the explanatory variables. We compared the Shannon diversity index and Richness using ANOVA and Kruskal–Wallis depending on normality^35^. In addition, we compared Shannon diversity index and Richness using ANOVA and Kruskal Wallis depending on normality^35^. For inferential analysis, we adopted a Poisson mixed effects regression in LME4 package in R using microbial core abundance as the outcome variable; HIV status, BMI, gender and microbial family as the explanatory variables; and sample identification (ID) as the random variable. Here we assessed the abundance at family level to minimise the number of covariate patterns. The families selected corresponded to the microbiota core membership across the sampling period to ensure equal representation during the participants’ visits to the clinic. To examine the dynamics across the three-sampling points, we analysed dataset A; for changes relative to baseline, we analysed datasets B and C (Fig. 1). For cumulative effect size of all the explanatory variables, we used PERMANOVA models with an Adonis function (9999 permutations) in phyloseq v1.26.0^36^ to identify microbiota structure-associations.

### Characterising the core and accessory microbiota

To determine the composition of the core and accessory microbiota^27,38^, we used the microbiome package in R https://github.com/microbiome/microbiome. To track changes in the core as proportion of richness across sampling points, we used genera that were common to all cores for the three sampling points i.e., baseline, months 2 and 5.

### Ethical approval and consent to participate

Ethical approval was provided by the Research and Ethics Committee of the School of Biomedical Sciences, Makerere University College of Health Sciences (reference #s SBS381/SBS542). Written informed consent was obtained from all the participants prior to enrolment into the study. All study methods were performed in accordance with the relevant guidelines and regulations.

## Supplementary Information

Supplementary Information 1.

Supplementary Information 2.

## Data Availability

All data generated or analyzed during this study are included in this published article (and its Supplementary Information files). The raw sequence data was deposited with links to BioProject accession number PRJNA564562 in the NCBI BioProject database https://www.ncbi.nlm.nih.gov/bioproject/.
