# Supplementary material for: Sputum microbiota profiles of treatment-naïve TB patients in Uganda before and during first-line therapy
Source: Sci Rep. 2021 Dec 29;11:24486. doi: 10.1038/s41598-021-04271-y (PMC8716532; doi:10.1038/s41598-021-04271-y)
Supplement: Supplementary file 2 — Supplementary Information 2. [file 41598_2021_4271_MOESM2_ESM.docx]

**Supplementary material:**

Sputum microbiota profiles of treatment-naïve TB patients in Uganda before and during first-line therapy

David Patrick Kateete^1§^, Monica M Mbabazi^1^, Faith Nakazzi^1^, Fred A Katabazi^1^, Edgar Kigozi^1^, Willy Ssengooba^2^, Lydia Nakiyingi^3,4^, Sharon Namiiro^5^, Alphonse Okwera^6^, Moses L Joloba^1^ and Adrian Muwonge^7§^

^1^Department of Immunology & Molecular Biology, School of Biomedical Sciences, Makerere University College of Health Sciences, Kampala, Uganda

^2^BSL-3 Mycobacteriology Laboratory, Department of Medical Microbiology, School of Biomedical Sciences, Makerere University College of Health Sciences, Kampala, Uganda

^3^Department of Medicine, School of Medicine, Makerere University College of Health Sciences, Kampala, Uganda

^4^Infectious Diseases Institute, Makerere University College of Health Sciences, Mulago Hospital Complex, Kampala, Uganda

^5^Makerere University Lung Institute, Makerere University College of Health Sciences, Kampala, Uganda

^6^TB Clinics, Mulago National Referral Hospital, Kampala, Uganda

^7^Division of Genetics and Genomics, Division of Infection and Immunity, The Roslin institute, University of Edinburgh, Edinburg, UK

^§^Contributed equally

Correspondence to david.kateete@mak.ac.ug & adrian.muwonge@roslin.ed.ac.uk

**Supplementary Figure S1:** Uploaded separately (see html file).

Map of Kampala and Wakiso districts showing geographical locations of the study participants. Adapted from ‘Leaflet’, an open-source JavaScript library for mobile-friendly interactive maps http://leafletjs.com/

**Supplementary Table S1:**

Comparison between *Mycobacterium* sequence counts and routine diagnostics for TB

| **Microbiota characteristic** | **Routine TB diagnostics** | | | | | |
| --- | --- | --- | --- | --- | --- | --- |
|  | AFB detection | | | LJ culture | | |
| *Mycobacterium* sequence reads | AFB+ | AFB- | Total* | Growth | No Growth | Total* |
| - Detected (%) | 55 (61) | 85 (86) | 140 | 52 (63) | 88 (83) | 140 |
| - Not detected (%) | 35 (39) | 14 (14) | 49 | 31 (37) | 18 (17) | 49 |
| Total | 90 | 99 | 189 | 83 | 106 | 189 |
|  | Estimate (95% CI) | | | Estimate (95% CI) | | |
| - Sensitivity (%) | 0.61 | (0.50, 0.71) | | 0.63 | (0.51, 0.73) | |
| - Specificity (%) | 0.14 | (0.08, 0.23) | | 0.17 | (0.10, 0.26) | |
| - Positive likelihood ratio | 0.71 | (0.59, 0.85) | | 0.75 | (0.63, 0.91) | |
| - Negative likelihood ratio | 2.75 | (1.59, 4.77) | | 2.20 | (1.33, 3.64) | |

*In this analysis, the number of samples exceeds the total number of patients (n=106) as it also includes treatment-response follow-up samples to determine sputum/culture conversion among the patients.

From this analysis, absence of *Mycobacterium* sequences is more than likely to predict an AFB negative result on microscopy (Ziehl-Neelsen [ZN] sputum smear staining), and no *Mycobacterium* growth on LJ culture but the reverse is not necessarily true. Furthermore, there is an inverse relationship between the number of *Mycobacterium* sequence counts and ZN sputum smear status for AFB detection, as well as *Mycobacterium* growth on LJ medium i.e., the detection of *Mycobacterium* sequences was associated with negative sputum smear status and negative *Mycobacterium* growth on LJ culture. The implication of this observation is not clear but could be due to the low yield of total bacterial DNA from AFB+ and LJ+ samples compared to the AFB- and LJ- samples. On the other hand, as respiratory infections are caused by microbial pathogens (mainly viruses and bacteria) that frequently interact with each other (reviewed by Bosch et al, reference #14), one can speculate that the growth/presence of *M. tuberculosis* is antagonistic to the growth/presence of certain bacteria or vice-versa. The Specificity, Sensitivity and other diagnostic parameters were estimated using epi.test function in the epiR package in R.

**Supplementary Figure S2:**

Comparison of microbiota sequence count, diversity and conventional diagnostics for TB

Supplementary Figure S2 shows how microbiota characteristics such as sequence count and diversity compare with outcomes on conventional TB diagnostics. Specifically, we show the relationship between sputum samples in which *Mycobacterium* sequences were detected and the diagnostic parameters routinely used in pulmonary TB i.e., bacillary load expressed as colony forming units (CFU) and AFB smear status. The top two panels (detection status of *Mycobacterium* sequences) depict sequence count per sample arranged in decreasing order and colored with CFU counts. It is apparent that the higher the CFU count, the lower the total DNA sequences a sample generates; the consequence of this has been expounded upon above (see Supplementary Table S1). The middle and bottom panels depict the same however, here it is Shannon diversity we compare; there is no discernible relationship between diversity and outcome on conventional TB diagnostics (i.e., CFU count and AFB smear status). However, there is an expected concordance between *Mycobacterium* read count and CFU count.

**Supplementary Table S2:**

Relationship between patient characteristics and sputum microbiota diversity as measured by PERMANOVA with adonis function (9,999 permutations) of weighted and unweighted UniFrac distances and Bray-Curtis dissimilarities

| **Parameter** | **Weighted UniFrac** | | | **Unweighted UniFrac** | | | **Bray-curtis** | | |
| --- | --- | --- | --- | --- | --- | --- | --- | --- | --- |
|  | Pseudo-F  Ratio | R^2^ | P value | Pseudo-F  Ratio | R^2^ | P value | Pseudo-F  ratio | R^2^ | P value |
| Sex | 0.28 | 0.011 | 0.8744 | 0.94 | 0.005 | 0.5047 | 1.29 | 0.007 | **0.0001** |
| Age | 1.60 | 0.008 | 0.1844 | 1.09 | 0.005 | 0.3045 | 1.20 | 0.006 | **0.0003** |
| District | 1.54 | 0.016 | 0.1478 | 1.17 | 0.012 | 0.1800 | 1.08 | 0.011 | **0.0106** |
| **BMI** | 0.75 | 0.008 | 0.6266 | 1.15 | 0.012 | 0.2101 | 1.09 | 0.011 | **0.0092** |
| Education | 0.39 | 0.002 | 0.7918 | 0.91 | 0.005 | 0.5577 | 1.10 | 0.006 | **0.0265** |
| **Nutritional status** | 1.45 | 0.007 | 0.2285 | 1.48 | 0.007 | 0.0705 | 1.23 | 0.006 | **0.0003** |
| **HIV status** | 0.94 | 0.005 | 0.4509 | 1.47 | 0.007 | 0.0679 | 1.12 | 0.006 | **0.0099** |
| **Sampling point** | 2.10 | 0.032 | 0.0224 | 2.60 | 0.039 | 0.0001 | 1.27 | 0.020 | **0.0001** |
| TB culture conversion | 0.25 | 0.001 | 0.7566 | 1.17 | 0.006 | 0.2280 | 0.97 | 0.005 | 0.7545 |
| TB lineage | 2.00 | 0.010 | 0.1020 | 0.98 | 0.005 | 0.4565 | 0.94 | 0.005 | 0.9148 |
| District | 2.07 | 0.021 | 0.0433 | 1.66 | 0.017 | 0.0085 | 1.05 | 0.011 | 0.0918 |

BMI, body mass index

This table shows the clinical covariate attributable effect size (R^2^), it allows us to partition the observed microbial structural variance. In this case we note that sampling point, which is our proxy for treatment, explains most of the explained variation i.e. %3.2, 3.9% and 2% respectively. Given that clinical covariates explain up to 13.6% of variance, this implies treatment accounts for 30% of the explained variance.

**Supplementary Figure S3:** Microbial diversity and distribution of diversity indices


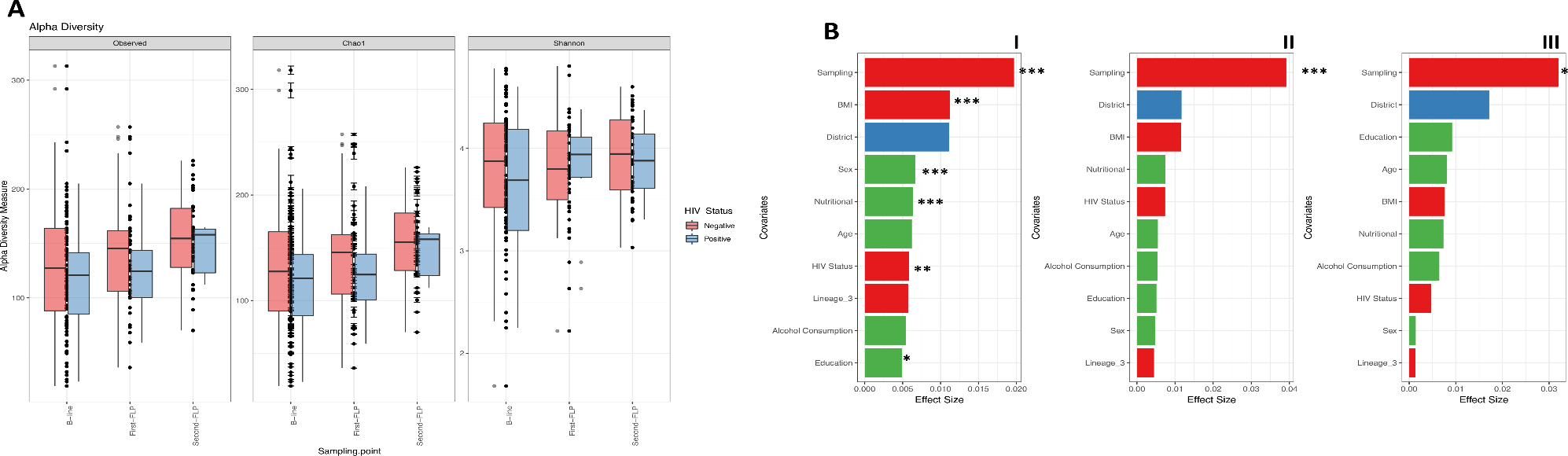


A

AA

B C

**Panel A** depicts the relationship between microbial diversity (based on three beta diversity indices) and participants’ covariates. The analysis was performed based on a PERMANOVA with Adonis function (9,999 permutations, see above – Supplementary Table S2). Note that the participants’ covariates are colored-coded as red (clinical), green (individual) and blue (geographical location/residence). Sub-panels I, II & III depict the effect size of each covariate with Bray-Curtis, Unweighted UniFrac & Weighted UniFrac distances as outcome variables, respectively. Asterisks depict covariates with statistically significant associations i.e., sampling point, body mass index (BMI), sex, nutritional status, HIV status, and education level. Sampling in this case is synonymous with temporal aspect which reflects anti-TB treatment.

**Panel B** depicts significant differences in sequence read counts vs. HIV status and *Mycobacterium* growth on cultures, while **Panel C** depicts alpha diversity (Shannon index) over time vs. HIV status. Here we observe significant differences in bacterial sequence count for HIV-positive patients with LJ-positive cultures (i.e., *Mycobacterium* growth) on the first- and second treatment follow-up visits (i.e., months 2 and 5, respectively).

**Supplementary Figure S4:**

Microbiota community overlap at OTU and genus levels at the three sampling points

A B

Microbiota community overlaps at OTU and genus levels (panels A and B, respectively) at the three sampling points – baseline (month 0), month 2 (First_FLP) and month 5 (Second_FLP).

Panel A shows that 3453, 1097 and 578 taxa (OTUs) are associated with pulmonary TB patients at baseline (month 0), month 2 and month 5, respectively, while panel B depicts the 178, 22 and 24 genera that are associated with patients at baseline (month 0), month 2 and month 5, respectively. Taking into account the treatment response outcomes and characteristics of patients during anti-TB therapy, these taxa/genera could be potential microbial signals for monitoring anti-TB treatment response. The related **Figure S5** (see below) is an extract of families and genera with the most viable abundance.

**Supplementary Figure S5:**

Bacterial families with high variability relative to HIV status


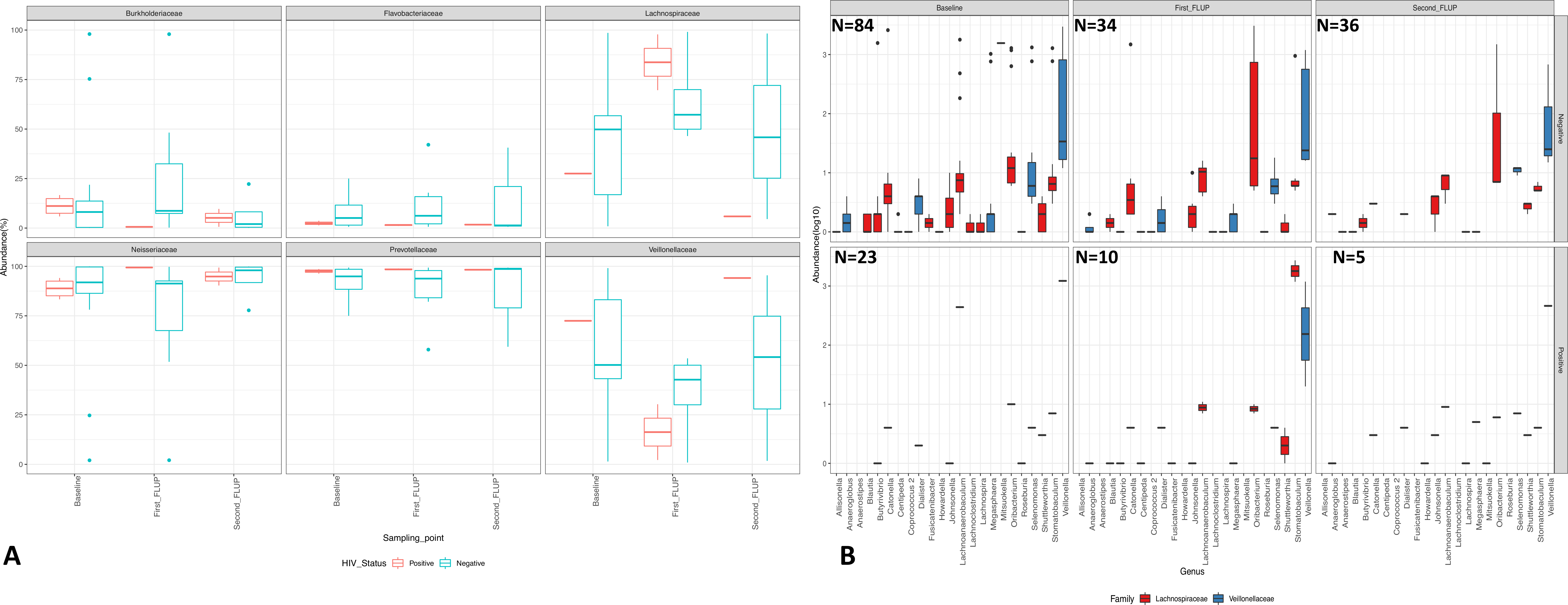


There were significant differences in total bacterial sequence counts vs. HIV status. **Figure S5** depicts six bacterial families i.e., *Burkholderiaceae*, *Flavobacteriaceae*, *Neisseriaceae*, *Prevotellaceae, Lachnospiracae and Veillonellaceae* with high variability during the sampling period. **Panel A** shows the relative abundance of the families relative to HIV status. The families with statistical significance in the distribution of sequences include *Burkholderiaceae*, *Flavobacteriaceae*, *Neisseriaceae* and *Prevotellaceae.* Altogether, there were fewer sequences for members of these families among HIV-positive patients compared to HIV-negative patients and by month 2 these families were nearly undetectable in HIV-positive patients. Of the six families, *Lachnospiracae* and *Veillonellaceae* had the highest variability relative to HIV status. **Panel B** shows which genera drive the variability observed in the two families, and *Alisonella, Catonella, Johnsonella*, and *Oribacterium* were exclusively detected in HIV-negative patients at months 2 and 5. Taken together, the differential distribution of the depicted taxa (families and/or genera) relative to HIV-status is of significance as these could be potential microbial indicators of disease progression in HIV-associated tuberculosis.

**Supplementary Figure S6:**

Sputum microbial community structure of all samples (n=106)

**Panel A** shows Alpha diversity analysis for indices like Richness, Simpson and Shannon. **Panel B** shows beta diversity analysis based on constrained ordination of the Bray-Curtis distances. Brown, orange and green represent the baseline (month 0) and treatment follow-up visits at months 2 and 3, respectively. The circular and triangular shapes denote HIV-negative and HIV-positive status, respectively. Note that this analysis is based on Datasets A, B and C of Figure 1 (see main text).

**Supplementary Figure S7:** **Sputum sample work-flow**


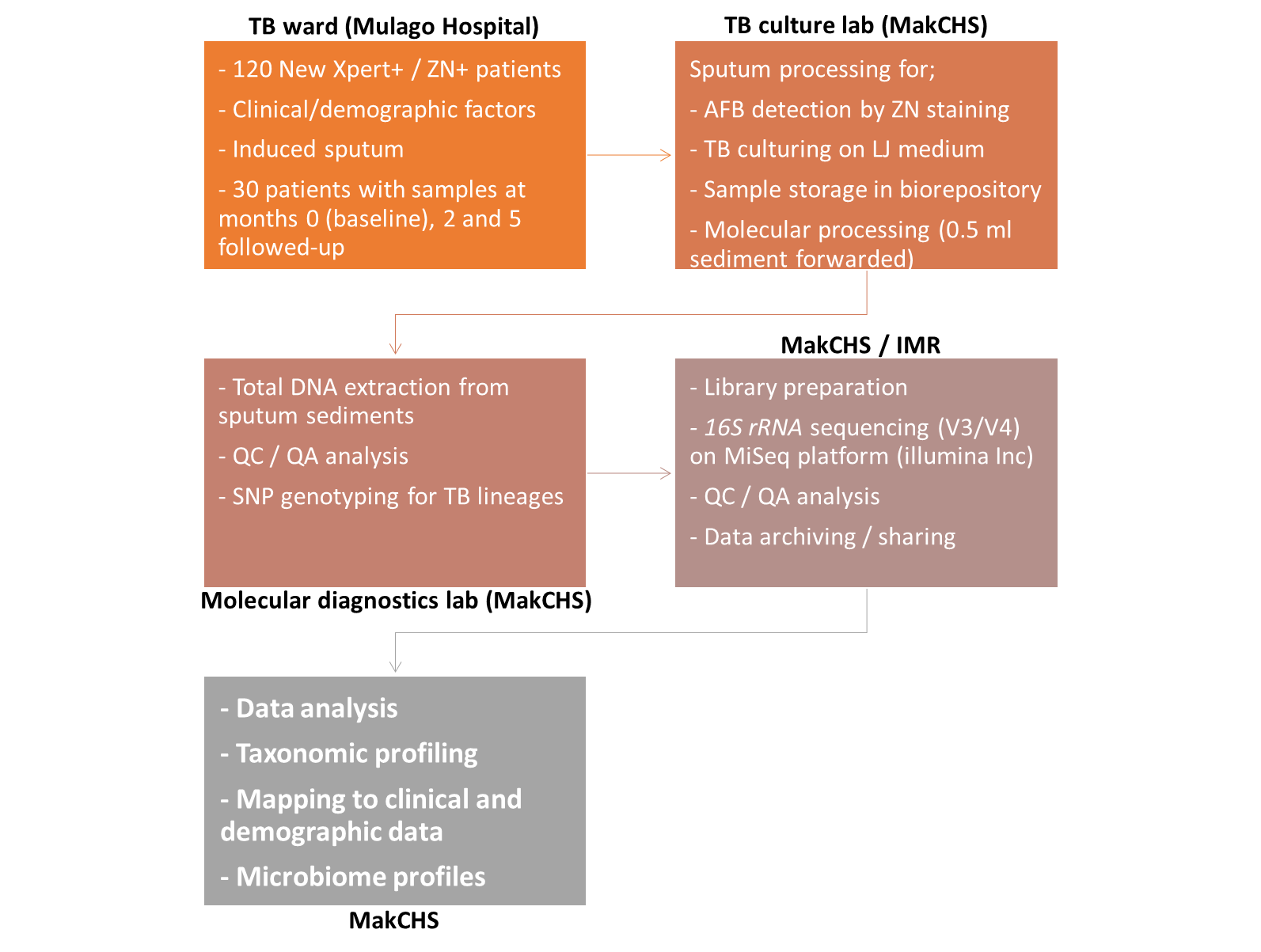


**Figure S7:** Depicts the sputum sample work-flow between the TB clinics at Mulago Hospital and the laboratories at Makerere University College of Health Sciences (MakCHS) where samples were processed and sequenced. For quality control (QC) / quality assurance (QA), samples were also sequenced at the IMR (Integrated Microbiome Resource), Dalhousie University, Canada. The TB clinics and Makerere laboratories are located on the same campus.
